# Giant enhancement of optical nonlinearity from monolayer MoS_2_ using plasmonic nanocavity

**DOI:** 10.1515/nanoph-2023-0714

**Published:** 2024-01-19

**Authors:** Liping Hou, Haosong Li, Qifa Wang, Xuetao Gan, Fajun Xiao, Jianlin Zhao

**Affiliations:** Key Laboratory of Light Field Manipulation and Information Acquisition, Ministry of Industry and Information Technology, and Shaanxi Key Laboratory of Optical Information Technology, School of Physical Science and Technology, Northwestern Polytechnical University, Xi’an 710129, China

**Keywords:** nanoparticles, plasmonic nanocavity, monolayer MoS_2_, second- and third-harmonic generations

## Abstract

The particle-on-mirror nanocavity, supporting multiple plasmonic resonances, provides an ideal platform to efficiently boost the nonlinear optical processes at the nanoscale. Here, we report on the enhancement of the second (SHG) and third-harmonic generations (THG) from the monolayer MoS_2_ using a multi-resonant Au nanosphere dimer-on-mirror nanocavity (DoMN). The strong plasmon hybridization between the dimer and underlying Au substrate leads to the emergence of two distinct cavity modes, which are intentionally aligned with the SH and TH frequencies, rendering a 15- and 68-fold enhancement for the SHG and THG of the monolayer MoS_2_, respectively. Further theoretical analysis yields that these remarkable nonlinearity enhancements are also ascribed to the amplification of nonlinear source because of the excellent spatial mode overlap and the high directivity of nonlinear emission enabled by the cavity modes. Our results pave the way for the implementation of low-cost, and highly efficient nonlinear photonics devices integrated with plasmonic nanocavities.

## 1 Introduction

Nanoscale nonlinear optical effects have greatly expanded the versatility of photonic applications ranging from the nonlinear optical modulator [1], [2] to nanolaser [3], super-resolution imaging [4], and biosensing [5]. However, the inherent inefficiency of nonlinear optical processes and limited active volume remain significant obstacles to the practical implementation of these applications [6], [7]. The plasmonic nanostructure possesses the capability to strongly confine light within the subwavelength volume [8], [9], making it an ideal candidate to enhance nonlinear optical processes, such as second (SHG) [10], [11], [12], [13] and third harmonic generations (THG) [14], [15]. Generally, the optical nonlinearity can be effectively enhanced by leveraging plasmonic resonances, which involve the amplification of fundamental light through strong local field interactions and/or the acceleration of nonlinear radiation via the antenna effect [14], [15], [16], [17]. In this regard, plasmonic metasurfaces fabricated by nanolithography techniques have been extensively employed to enhance the SHG of nonlinear optical materials such as two-dimensional transition metal dichalcogenides (TMDs) [18], [19], [20], [21], [22]. In particular, metasurfaces with the double-resonant feature exhibit the capability to boost the fundamental excitation and nonlinear emission simultaneously, delivery a considerable high SHG conversion efficiency [10], [23]. Nevertheless, the complex structural designs of metasurfaces typically require sophisticated and costly lithography procedures, imposing a restriction on optical nonlinearity enhancements.

Alternatively, the recently emerging particle-on-mirror (PoM) plasmonic nanocavity [24], [25], [26], [27], comprising a closely spaced nanoparticle and an ultrasmooth metal film, provides an elegant and cost-effective platform for enhancing the optical nonlinearity [28], [29]. The combination of the thin film deposition technique and the bottom–up approach enables the precise control of nanocavity size even down to the subnanometer level [30], [31], [32]. This also facilitates the integration of nonlinear optical nanomaterials such as quantum dots and TMDs [33], [34], [35], [36]. Therefore, the weak signal of nonlinear processes can be significantly magnified due to the ultrasmall mode volume and exceptional local field enhancement provided by nanocavities [33], [34]. In addition, the colloidal synthesis technique enables precise control over the size and shape of nanoparticles, offering a straightforward way to spectrally align the resonance of PoM nanocavity with the optical nonlinear processes [37], [38], [39]. Recent studies have also demonstrated that the strong plasmon–exciton coupling in TMDs-PoM hybrid systems can produce an extremely large effective second-order susceptibility in TMDs-PoM hybrid systems, rendering a 3000-fold increase in SHG intensity [40]. Besides, the deliberate selection of mode types within the PoM nanocavity allows electromagnetic symmetry-breaking induced at the SH frequency, thereby resulting in efficient and high-directional SHG emissions [28]. Of note, to expand the operation wavelength of nanophotonic devices and applications [41], it is imperative to simultaneously enhance both the SHG and THG in the hybrid PoM nanocavity system [34]. However, this area still remains largely unexplored.

To this end, we propose an Au nanosphere dimer-on-mirror nanocavity (DoMN) to enhance the SHG and THG of monolayer molybdenum disulfide (MoS_2_). In contrast to the single-particle nanocavity configuration, the plasmon hybridization between nanoparticles and their mirror images facilitates the generation of multiple cavity modes within the visible and near-infrared range, which can be readily adjusted to match both the SH and TH frequencies. This harmonic resonance strategy together with the merit of spatial mode overlap and high emission directivity results in a giant enhancement of SHG and THG intensities from the monolayer MoS_2_. Our findings present a straightforward and feasible approach to concurrently enhance the SHG and THG of monolayer MoS_2_, thereby paving the way for high-efficiency integrated nonlinear devices.

## 2 Experimental and simulation details

### 2.1 Sample fabrication

The Au nanosphere dimer and Au film were chosen as building blocks for the construction of the plasmonic nanocavity using the bottom-up assembly technique. Specifically, a 50-nm-thick Au film was deposited onto the silicon substrate via thermal evaporation at a deposition rate of 2 Å/s. The Au film was transferred onto the SiO_2_/Si substrate by a template-stripping method, rendering atomic flatness with a roughness of approximately 0.26 nm. The alumina layer, with a thickness of 5 nm, was subsequently grown onto the Au film using the atomic layer deposition technique at an operating temperature of 150 °C. The monolayer MoS_2_ was mechanically exfoliated from bulk crystal (HQ-graphene, Inc.), and then transferred onto the Al_2_O_3_/Au substrate with the dry transfer method. Finally, 150-nm-diameter Au nanospheres (Nanoseedz, Inc.), encapsuled with a 2 nm cetyltrimethylammonium bromide (CTAB) surfactant polymer, were spin-coated onto the monolayer MoS_2_ to form a MoS_2_-DoMN hybrid structure. It yields a nanosphere density of 0.06/μm^2^ on the MoS_2_ with an optimized separation larger than 2 μm to ensure the single-particle-level measurement.

### 2.2 Optical characterization

The scattering spectra of the MoS_2_-DoMNs were measured using a home-built dark-field confocal microscope, where the sample was illuminated with polarization-controlled white light at an incident angle of 60°. The scattering signal was collected with a 50× objective (Nikon, NA0.6). After spatially filtering with a pinhole, the scattered signal was directed to either a CCD-equipped spectrometer (Andor Shamrock SR-303IB) for spectral analysis or a CCD camera for dark-field imaging. The SHG and THG measurements were conducted using a custom-built nonlinear optical microscopy system (see Supplementary Material, Figure S1). A fiber-based pulsed laser (80 fs, 80 MHz, *λ* = 1550 nm) was focused onto the samples through a 100× objective (Leica, NA0.9) with a focal spot size of ∼1 μm. The nonlinear signals were collected by the same objective. After passing through a dichroic mirror, the collected SHG and THG signals were delivered to the spectrometer (HORIBA *i*HR320) for spectral analysis. Notably, the target dimer was first located from the SEM image, as shown the inset of Figure S2(A). By taking the edges of MoS_2_ flake as spatial markers, the same dimer can be identified in the dark-field image. To eliminate undesired signal contributions, dimers located at a minimum separation of 2 μm from other nanosphere clusters and the MoS_2_ edges were intentionally chosen in linear and nonlinear optical measurements through the pinhole.

### 2.3 Numerical simulation

The linear responses of the MoS_2_-DoMNs were calculated using the finite element method (FEM). The geometries of the MoS_2_-DoMNs were modeled to match their experimental counterparts. The refractive index of the Al_2_O_3_ spacer and CTAB surfactant polymer were set to be 1.5 and 1.435, respectively. The permittivity of Au was taken from the experimental data of Johnson and Christy [42]. The monolayer MoS_2_ was modeled as a film with a thickness of 1 nm. To accurately describe the dielectric properties of MoS_2_, the mesh size of the film was controlled to be below 0.2 nm, and its wavelength-dependent optical constant was obtained from the experimental data reported in Ref. [43]. For the scattering calculations, we initially employed a plane wave with an incident angle of 60° to produce the background electric field in the absence of a dimer. Subsequently, this calculated field was utilized as an excitation to derive the scattering field in the presence of the dimer. According to Gauss’s law, the charge distribution of the nanocavity was determined by evaluating the disparity in the normal component of the electric field above and below the metallic surface. The SHG and THG of the MoS_2_-DoMNs were calculated using a two-step method: first, the second- and third-order polarizations were derived from the linear response of the fundamental field according to **P** ^(2*ω*)^ = *χ* ^(2)^ **E**(*ω*)**E**(*ω*) and **P** ^(3*ω*)^ = *χ* ^(3)^ **E**(*ω*)**E**(*ω*)**E**(*ω*) [44], [45], [46], respectively. Then, **P** ^(2*ω*)^ and **P** ^(3*ω*)^ are regarded as nonlinear sources, which radiate the SHG and THG fields in the far-field domain (see Supplementary Material).

## 3 Results and discussion

**Figure 1:**
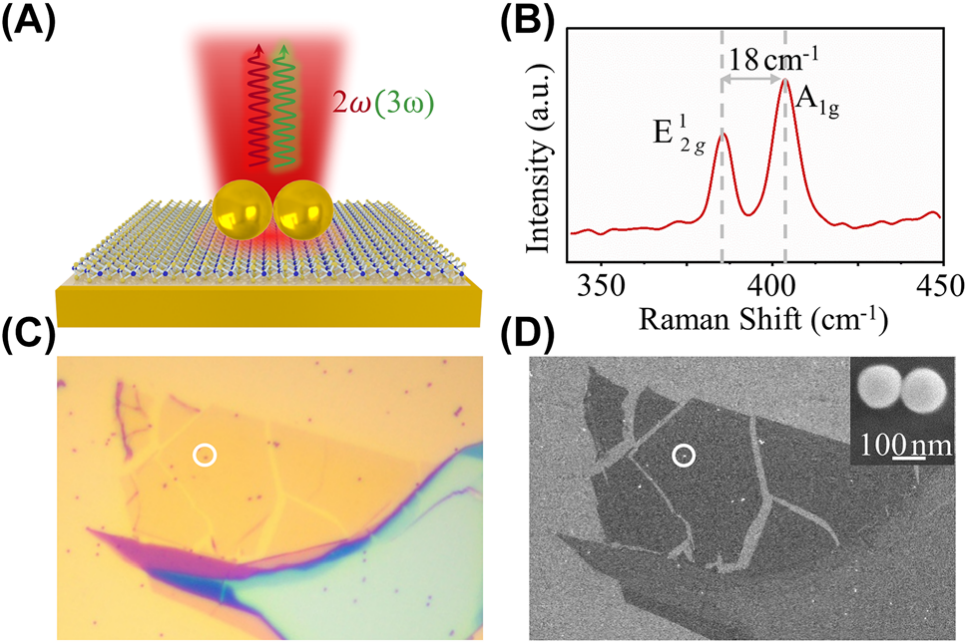
Hybrid structure of the monolayer MoS_2_ coupled Au nanosphere dimer-on-mirror nanocavity (DoMN). (A) Schematic of the monolayer MoS_2_-DoMN. (B) Raman spectrum of the monolayer MoS_2_ taken nearby the dimer. (C) Bright- and (D) SEM images of the MoS_2_-DoMN. The white circles indicate the location of MoS_2_-DoMN. The inset of (D) shows the Au nanosphere dimer, where the scale bar is 100 nm.

The MoS_2_-DoMN hybrid structure is schematically depicted in Figure 1(A), which consists of an Au nanosphere dimer on top, an ultrasmooth Au film, and a monolayer MoS_2_. The monolayer MoS_2_ was identified by Raman spectroscopy carried out on a commercial confocal microscope (WITec, Alpha 300R). The interval between the out-of-plane mode A_1g_ and in-plane mode E^1^_2g_, as shown in Figure 1(B), is observed to be 18 cm^−1^, consistent with the typical mode interval in the monolayer MoS_2_ [47]. The bright field and scanning electron microscope (SEM) images of the hybrid structure are, respectively, displayed in Figure 1(C) and (D), where the Au dimer is marked within the white circles. The Au nanosphere radius is determined to be ∼75 nm using SEM, as clearly depicted in the inset of Figure 1(D).

**Figure 2:**
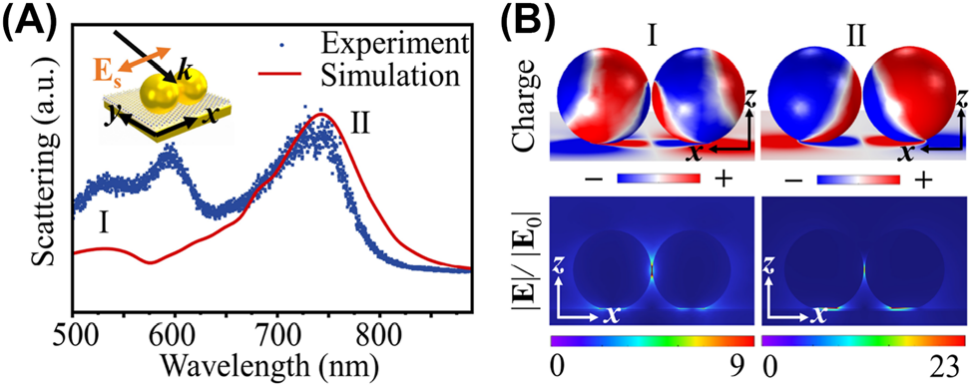
Plasmonic response of MoS_2_-DoMNs. (A) Measured (dots) and simulated (solid line) scattering spectra of MoS_2_-DoMNs. The inset depicts the excitation configuration used in the measurement. (B) Charge distributions (upper panels) and electric field enhancement maps (lower panels) of modes I and II as labeled in (A).

The multi-resonance characteristic of MoS_2_-DoMN is revolved using the dark-field scattering spectroscopy, where the side-illumination is polarized along the dimer axis, i.e. an s-polarized beam as depicted in the inset of Figure 2(A). The scattering spectrum of the hybrid structure, as shown blue dots in Figure 2(A), exhibits 3 pronounced plasmonic modes spanning the visible and near-infrared range. This multi-resonance feature is also well reproduced by FEM simulations, as evidenced by the red solid line in Figure 2(A). To elucidate the origin of the plasmonic modes observed in Figure 2(A), we calculate the transient surface charge distributions of the hybrid structure. Here, we focus on the charge maps of mode I (∼530 nm) and II (∼750 nm), whose resonance bands can cover the SH and TH frequencies generated by the 1550 nm fundamental beam. As observed in the upper panels of Figure 2(B), the s-polarized beam induces a pronounced longitudinal plasmon coupling between two nanospheres, resulting in the formation of the bonding electric quadrupole and dipole modes in the dimer. These bonding plasmonic modes couple with their mirror-image charges in the underlying Au film, leading to the emergency of cavity modes I and II, in accordance with prior observations in the Refs. [48], [49], [50]. It should be noted that due to the asymmetry of the DoMN configuration, plasmon couplings between the dimer and Au film contribute to the nonvanishing resultant dipole moments, exhibiting considerable scattering ability as evidenced by spectral peaks in Figure 2(A). In addition, the DoMN configuration can lead to a significant reduction in radiation loss [48], [51]. Consequently, when the TH and SH wavelengths fall within the modes I and II range, the localized nonlinear source can be effectively transformed to the far-field nonlinear emission through the coupling with the resultant dipole. The low panels of Figure 2(B) show the near-field distributions of the hybrid system, which have 9- and 23-folds of field enhancement at the MoS_2_ plane for modes I and II, respectively. Therefore, besides the acceleration of the nonlinear radiation rate, modes I and II are expected to further augment the nonlinear signals by amplifying the TH and SH nonlinear sources.

**Figure 3:**
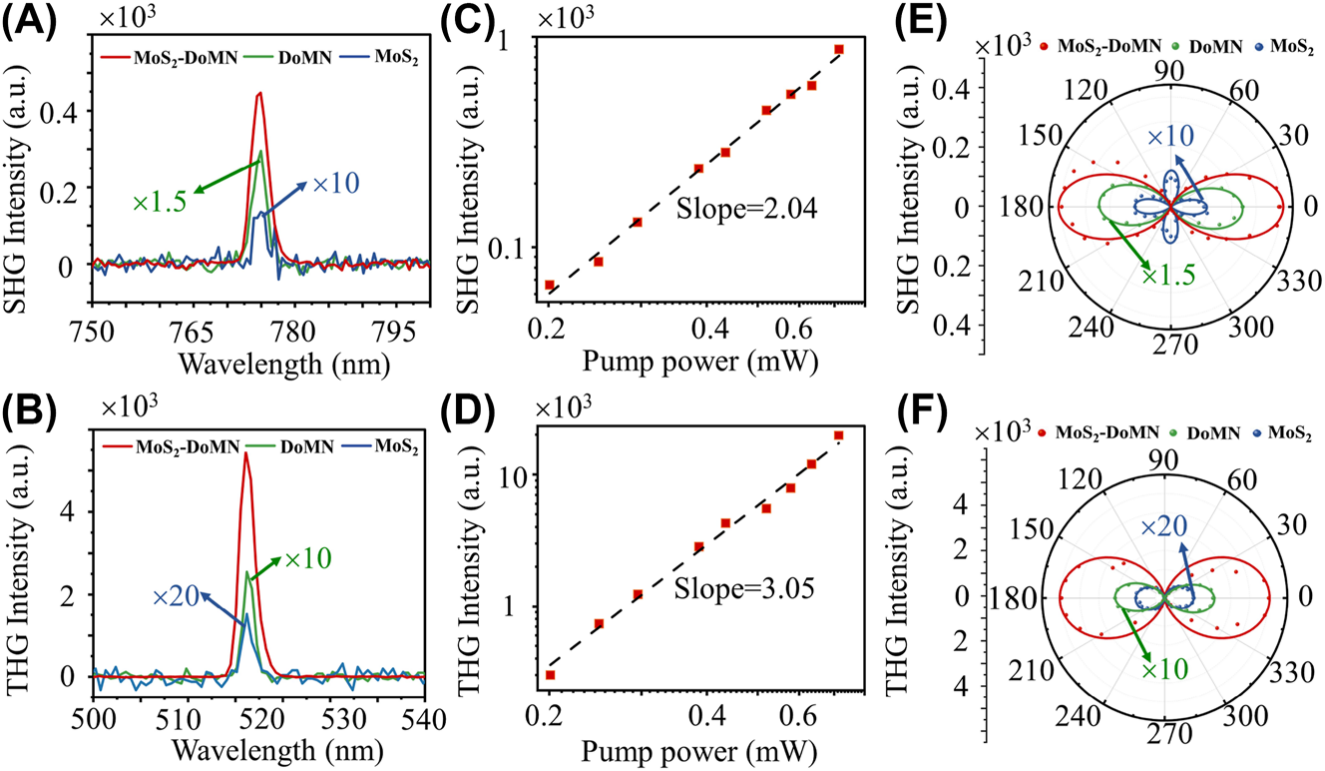
Characterization of nonlinear response in hybrid structures. (A) SHG and (B) THG spectra of MoS_2_-DoMN (red), DoMN (green), and MoS_2_ on SiO_2_/Si substrate (blue) under an excitation wavelength of 1550 nm. (C) SHG and (D) THG peak intensities as a function of laser power in a log-log plot. Polar plots of the polarization of (E) SHG and (F) THG signals from the monolayer MoS_2_ on SiO_2_/Si substrate (blue dots), DoMN (green dots), and the MoS_2_-DoMN (red dots). The experimental results are well-fitted by the theoretical model (solid lines).

Subsequently, we measured the nonlinear optical responses of the MoS_2_-DoMN hybrid system. Here, the 1550 nm fundamental beam is kept at a constant power of 0.5 mW and polarization along the dimer axis. As expected, due to the frequencies of nonlinear emissions overlapped with the nanocavity resonances, a prominent THG and SHG signals are observed as shown the red lines in Figure 3(A) and (B). As comparisons, we perform the nonlinear measurement on the bare DoMN structure and the monolayer MoS_2_ on the SiO_2_/Si substrate, depicted as the green and blue lines in Figure 3(A) and (B). After subtracting the contribution from the DoMN structure, we define the effective enhancement factor (EF) as

(1) $$\text{EF}=\frac{I_{\dim}-I_{0}-I_{\dim0}}{I_{0}}$$

where *I*_dim_, *I*_0_, and *I*_dim0_ are the SHG/THG intensities of the MoS_2_-DoMN hybrid structure, pristine monolayer MoS_2_, and the DoMN, respectively. According to Eq. (1), the enhancement factors are determined to be 15- and 68-folds for the SHG and THG of MoS_2_, respectively. We further measure the SHG and THG from 3 other MoS_2_-DoMN hybrid structures, yielding an averaged enhancement of 13-fold and 65-fold for SHG and THG, respectively (see Supplementary Material). The enhancements of the SHG and THG from TMDs using diverse nanostructures are summarized in Table S2. Considering that the collected nonlinear signals also include contributions from the background MoS_2_, the actual nonlinear responses of MoS_2_ enhanced by the DoMN should be large. We discussed the effective enhancement factor in the Supplementary Material. Figure 3(C) and (D), respectively, present the log-log plots of SHG and THG signals from MoS_2_ in the cavity as a function of the pumping power. The linear fits to these two power-dependent evolutions reveal slopes of ∼2 and ∼3, confirming the second- and third-order nature of SHG and THG nonlinear processes.

The polarization-resolved analysis of plasmon-enhanced SHG and THG emissions is further investigated, as shown in Figure 3(E) and (F). We fixed the analyzer parallel to the dimer axis, while the polarization of fundamental light is rotated in a step of 5° using a half-wave plate. Because of the *D*_3h_ crystal symmetry [52], the SHG of the monolayer MoS_2_ on SiO_2_/Si substrate displays a 4-fold symmetry which is in accordance with the theoretical predictions (also see the blue line in Figure 3(E)). The polarization-dependent THG is independent on the crystal orientations of the monolayer TMDs [53], thus, showing a 2-fold symmetry for the pristine MoS_2_, consistent with the theoretical model (see Supplementary Material). Whereas the polar plot of SHG and THG from bare DoMN (green dots) and MoS_2_-DoMN (red dots) all exhibit similar two lobe pattern. The reason lies in the enhanced optical nonlinearities for both DoMN and MoS_2_-DoMN can be attributed to the amplification of nonlinear sources by the cavity modes I and II. The scattering spectra in Figure S4(A) demonstrate both cavity modes I and II have the same polarization dependent behavior, with the peak intensity following the two-fold symmetry (see Figure S4(B) and (C)). Consequently, the polar diagrams of nonlinear emissions from both the bare DoMN and hybrid structure exhibit two-fold symmetry patterns. These patterns show maximum intensity at 0° (180°) and 90° (270°) when cavity modes I and II are strongest or absent, respectively. These polarization-dependent features of the hybrid structure are also well described by the theoretical model (see Supplementary Material), as shown the red line in Figure 3(E) and (F).

**Figure 4:**
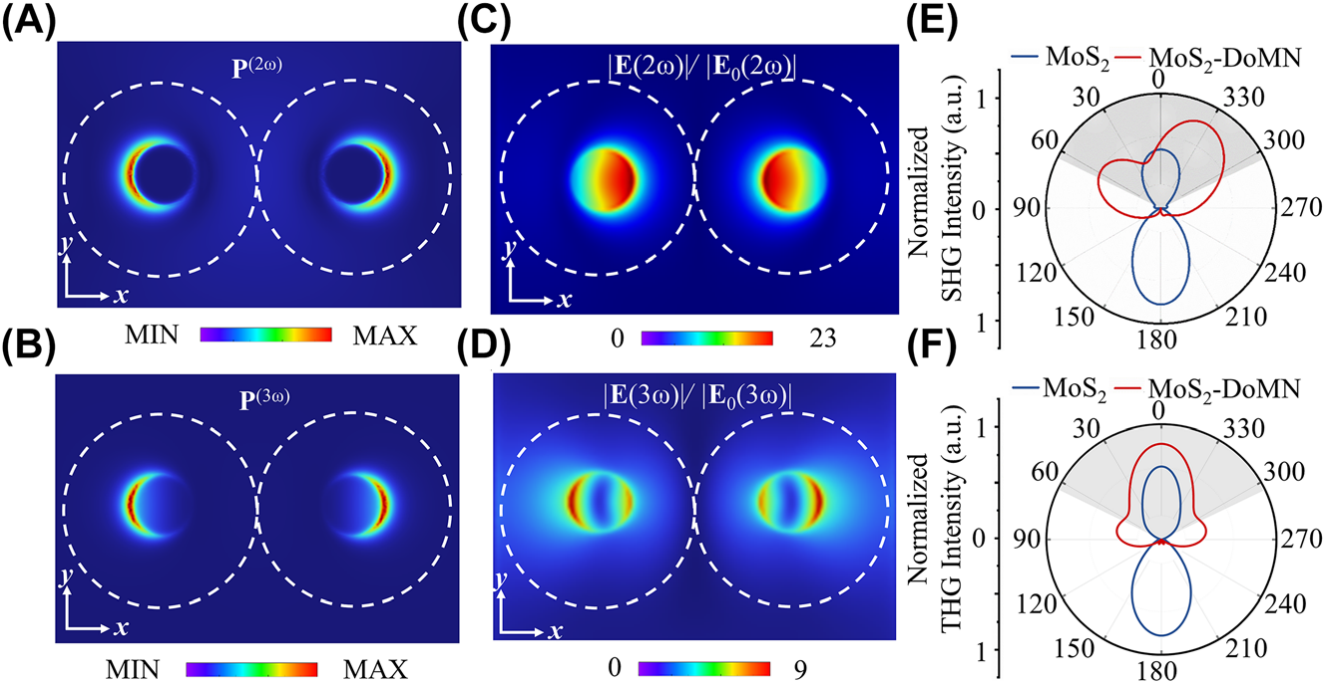
Nonlinear polarization distributions at (A) SH and (B) TH frequencies. Field enhancement maps of the MoS_2_-DoMN at the (C) SH and (D) TH frequencies. Simulated far-field radiation patterns of (E) SHG and (F) THG from the MoS_2_-DoMN (red curves) and MoS_2_ (blue curves) in the *xz*-plane. The gray area indicates the collection cone of the objective.

To further unravel the role of cavity modes in the nonlinearity enhancement of monolayer MoS_2_, we calculate the near-field distribution of nonlinear polarization as well as their far-field radiation. As shown in Figure 4(A) and (B), both the second- and third-order polarizations exhibit crescentic distributions around the outer periphery of the touching points between the dimer and substrate. These nonlinear polarizations show a good spatial overlap with the field enhancement maps produced by cavity modes I and II at the plane of MoS_2_, as illustrated in Figure 4(C) and (D). Especially, the third-order polarization is localized precisely at the field maximum of cavity mode I. Therefore, the large SHG and THG enhancement can be ascribed to the substantial amplification of nonlinear sources by leveraging cavity modes, due to the excellent spatial mode matching. The far-field emission patterns of SHG and THG signals at the *xz* plane are presented in Figure 4(E) and (F), where the grey shaded areas indicate the collection angular of the objective (NA = 0.9). Of note, the coherent overlap between SHG from MoS_2_ and DoMN has a destructive interference at the up-left zone and a constructive interference and up-right zone (Figure S5), leading to an asymmetric SHG radiation of the MoS_2_-DoMN shown in Figure 4(E). As seen, the nonlinear signals from the SiO_2_/Si-supported MoS_2_ mainly radiate to the substrate side. In contrast, in virtue of the high radiation directivity of modes I and II, the SHG and THG of MoS_2_-DoMN are predominantly governed by the backward emissions, resulting in 1.96- and 2-fold increases of collection efficiency for SHG and THG, respectively.

## 4 Conclusions

In summary, we have proposed a method to simultaneously enhance the second (SHG) and third harmonic generations (THG) of monolayer MoS_2_ using a multi-resonant Au nanosphere dimer-on-mirror nanocavity (DoMN). Combining the dark-field scattering spectroscopy and full-wave simulations reveals two profound film-coupled plasmonic bonding modes in the visible and near-infrared range. These two cavity modes have a good frequency match with the SH and TH frequencies, meanwhile, ensuring an excellent spatial overlap between the nonlinear polarizations and field enhancements. These virtues work with the high emission directivity of cavity modes, delivering 15- and 68-fold of enhancement for SHG and THG of monolayer MoS_2_, respectively. The exceptional SHG and THG performances imply the promising prospect of our MoS_2_-NoMN hybrid structure in nonlinear optical applications.

## Supplementary Material

Supplementary Material Details
