# Supplementary material for: Giant enhancement of optical nonlinearity from monolayer MoS2 using plasmonic nanocavity
Source: Nanophotonics. 2024 Jan 19;13(3):349–55. doi: 10.1515/nanoph-2023-0714 (PMC11502082; doi:10.1515/nanoph-2023-0714)
Supplement: Supplementary file 1 — Supplementary Material Details [file j_nanoph-2023-0714_suppl_001.docx]

Supplementary material

**Giant enhancement of optical nonlinearity from monolayer MoS_2_ using plasmonic nanocavity**

Liping Hou,^1^ Haosong Li,^1^ Qifa Wang,^1^ Xuetao Gan, ^1, *^ Fajun Xiao^1, *^ and Jianlin Zhao^1, *^

^1^ Key Laboratory of Light Field Manipulation and Information Acquisition, Ministry of Industry and Information Technology, and Shaanxi Key Laboratory of Optical Information Technology, School of Physical Science and Technology, Northwestern Polytechnical University, Xi’an 710129, China

^*^ Correspondence E-mail: [xuetaogan@nwpu.edu.cn](mailto:xuetaogan@nwpu.edu.cn); [fjxiao@nwpu.edu.cn](mailto:fjxiao@nwpu.edu.cn); [jlzhao@nwpu.edu.cn](mailto:jlzhao@nwpu.edu.cn).

**Content**

S1. Optical setup for nonlinear optical measurements.

S2. SEM and dark-field images of the MoS_2_-DoMN.

S3. Calculations of SHG and THG from the monolayer MoS_2_-DoMN.

S4: SHG and THG from 3 other MoS_2_-DoMN hybrid structures.

S5. Effective enhancement factor.

S6. Polarization-resolved scattering spectra.

S7: SHG far-field amplitude patterns; phase differences (|*ϕ*_MoS2_-*ϕ*_DoMN_|) of the SHG far-field between MoS_2_ and DoMN in the *xz*-plane.

S8. Calculation of the parallel components of the SHG and THG intensities.

S1: Optical setup for nonlinear optical measurements

**
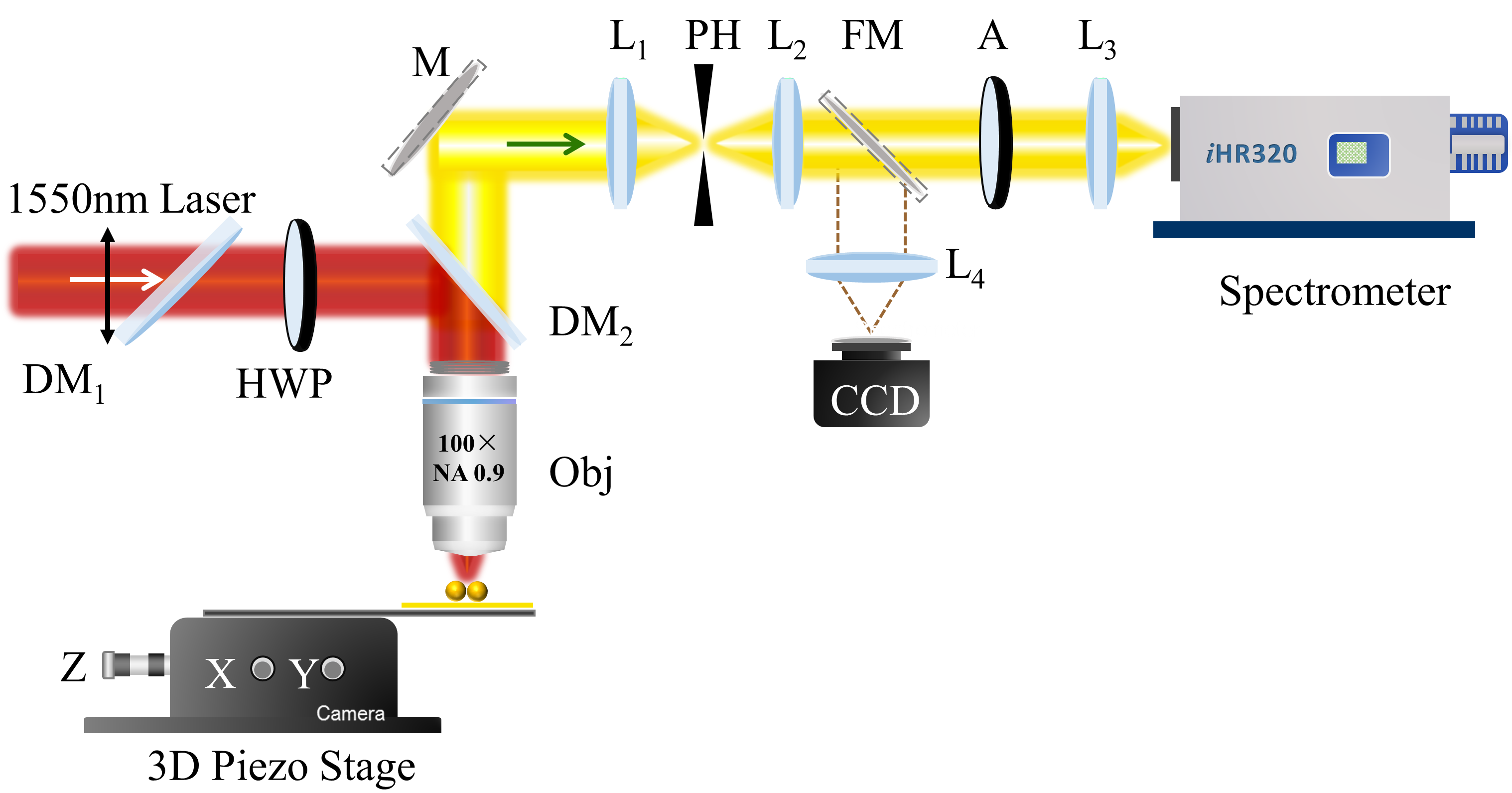
**

**Figure S1:** Optical setup for nonlinear optical measurements. L: lens, HWP: half-wave plate, PH: pinhole, DM: dichroic mirrors, FM: flip-mirror, A: analyzer, Obj: objective lens.

S2. SEM and dark-field images of the MoS_2_-DoMN.


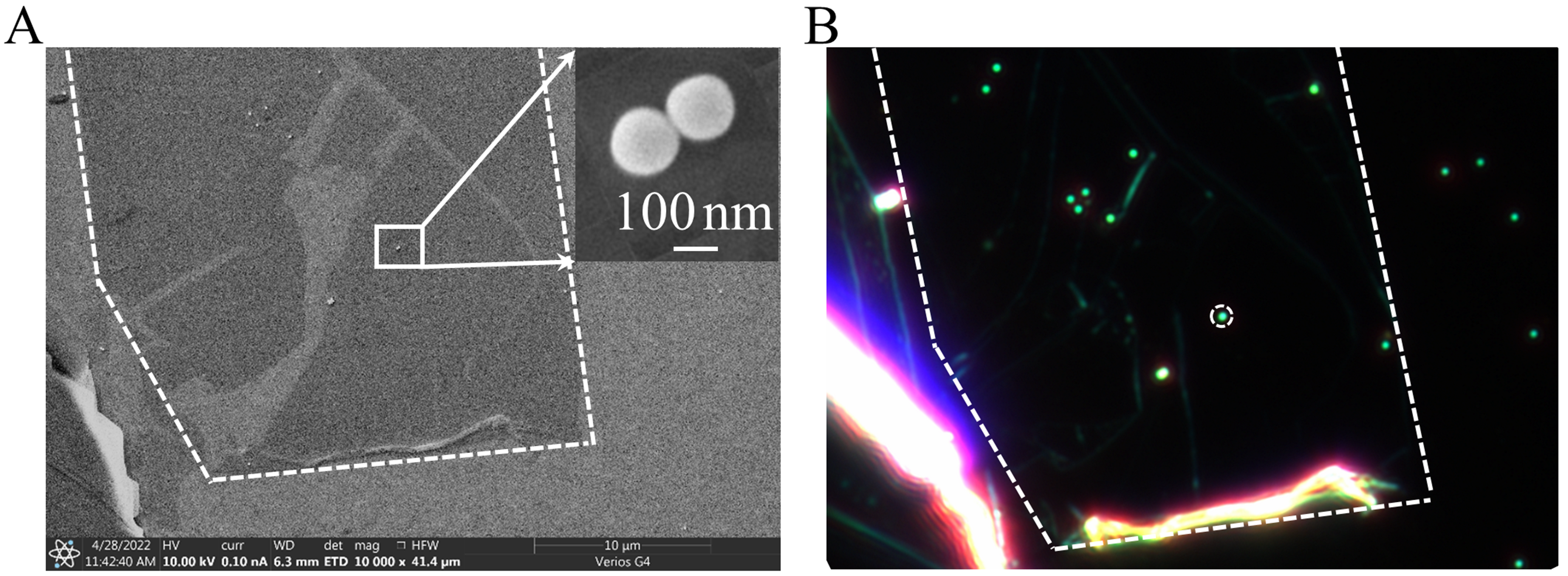


**Figure S2:** (A) SEM and (B) dark-field images of the MoS_2_-DoMN. The dashed circle indicates actual detection area. The inset of (A) shows the zoom-in SEM image of Au nanosphere dimer, where the scale bar is 100nm.

S3. Calculations of SHG and THG from the monolayer MoS_2_-DoMN.

The initial step involved in determining the field distribution $\mathbf{E}\left( r,\omega\right)$ of MoS_2_-DoMN at the fundamental frequency using COMSOL based on the finite element method. Subsequently, the second- and third-order nonlinear polarizations of the monolayer MoS_2_ and DoMN were derived from $\mathbf{P}^{(2)}\left( r,2\omega\right)=\chi^{(2)}\mathbf{E}\left( r,\omega\right)\mathbf{E}\left( r,\omega\right)$ and $\mathbf{P}^{(3)}\left( r,3\omega\right)=\chi^{(3)}\mathbf{E}\left( r,\omega\right)\mathbf{E}\left( r,\omega\right)\mathbf{E}\left( r,\omega\right)$. Considering the monolayer MoS_2_ belongs to the $\text{D}_{\text{3h}}$ point group, $\chi^{(2)}$ possesses a single non-zero element $\chi^{(2)}\equiv\chi_{yyy}^{\left( 2 \right)}={-\chi}_{xxy}^{\left( 2 \right)}=-\chi_{xyx}^{\left( 2 \right)}=-\chi_{yxx}^{\left( 2 \right)},$ and $\chi^{(3)}$comprises the non-zero elements as follows: $\chi_{yyyy}^{\left( 3 \right)}=\chi_{xxxx}^{\left( 3 \right)}=\chi_{yyxx}^{\left( 3 \right)}+\chi_{yxxy}^{\left( 3 \right)}+\chi_{yxyx}^{\left( 3 \right)},\chi_{yyxx}^{\left( 3 \right)}=\chi_{xxyy}^{\left( 3 \right)},$ ${\chi_{xyyx}^{\left( 3 \right)}=\chi}_{yxxy}^{\left( 3 \right)},$ and ${\chi_{xyxy}^{\left( 3 \right)}=\chi}_{yxyx}^{\left( 3 \right)}$. Assuming the excitation with arbitrary polarization $\mathbf{E}\left( r,\omega\right)=\left| \mathbf{E}\left( r,\omega\right) \right|\left[ \vec{x}cos(\theta)\pm i\vec{y}\sin(\theta) \right]$, the second- and third-order nonlinear polarization of monolayer MoS_2_ can be described by

$\mathbf{P}_{\mathrm{MoS}_{2}}^{(2)}\left( r,\omega\right)=\varepsilon_{0}\chi_{yyy}^{\left( 2 \right)}\left| \mathbf{E}\left( r,\omega\right) \right|^{2}\left[ \mp\sin\left( 2\theta\right)\vec{x}-\vec{y} \right],$ (1)

and

$\mathbf{P}_{\mathrm{MoS}_{2}}^{(3)}\left( r,\omega\right)=\varepsilon_{0}\chi_{yyyy}^{\left( 3 \right)}\left| \mathbf{E}\left( r,\omega\right) \right|^{3}\left[ \vec{x}cos(\theta)\pm i\vec{y}\sin(\theta) \right]\cos\left( 2\theta\right),$ (2)

where $\varepsilon_{0}$is the vacuum permittivity, $\chi_{yyy}^{\left( 2 \right)}$ and $\chi_{yyyy}^{\left( 3 \right)}$are taken from the experimental data as $2.2 \mathrm{pmV}^{-1}$and ${{10}^{-17} m}^{2}V^{-2}$ [1]，respectively. For the metallic part of the hybrid structure, the second-order surface susceptibility of Au is dominated by the component $\chi_{\perp\perp\perp}^{\left( 2 \right)}$. As a result, the corresponding nonlinear polarization is written as

$\mathbf{P}_{\mathrm{Au}}^{(2)}\left( r,\omega\right)=\chi_{\perp\perp\perp}^{\left( 2 \right)}\mathbf{E}_{\perp}\left( r,\omega\right)\mathbf{E}_{\perp}\left( r,\omega\right)\mathbf{E}_{\perp}\left( r,\omega\right)$, (3)

where$\chi_{\perp\perp\perp}^{\left( 2 \right)}$ was set as $40 \mathrm{pmV}^{-1}$ [2]. The general expression for the third-order nonlinear polarization of Au structure is denoted as

$\mathbf{P}_{\mathrm{Au}}^{(3)}\left( r,\omega\right)=\chi_{\mathrm{Au}}^{\left( 3 \right)}\mathbf{E}\left( r,\omega\right)\mathbf{E}\left( r,\omega\right)\mathbf{E}\left( r,\omega\right),$ (4)

where $\chi_{\mathrm{Au}}^{\left( 3 \right)}$ was taken as ${3.4{\times10}^{-16} m}^{2}V^{-2}$ [3].The nonlinear polarizations calculated from Eq. (1)-(4) were then considered as the SHG and THG sources of the MoS_2_-DoMN hybrid structure, which was executed by the finite element solver to obtain the nonlinear signals at TH and SH frequencies. The radiation patterns were derived using the far-field domain module.

S4: SHG and THG from 3 other MoS_2_-DoMN hybrid structures.


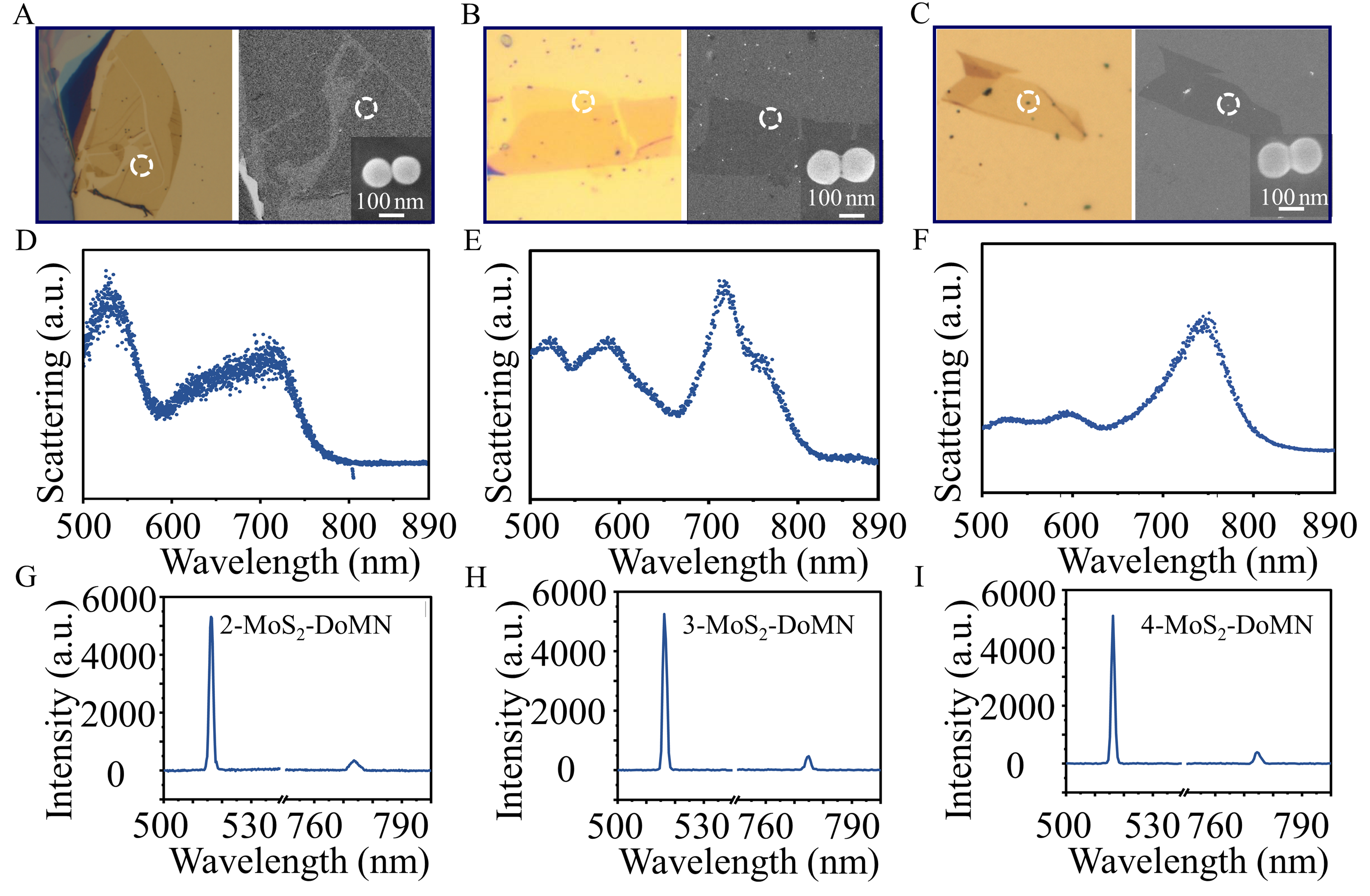


**Figure S3:** (A)-(C) Full views of bright-field (left panels) and SEM (right panels) for the three MoS_2_-DoMN hybrid structures. The inset of (right panels) shows the Au nanosphere dimer, where the scale bar is 100 nm. (D)-(F) Measured scattering spectra, (G)-(I) SHG and THG spectra of the three MoS_2_-DoMNs.

**Table S1:** Summary of SHG and THG EF.

| MoS_2_-DoMN | | 1 | 2 | 3 | 4 | averaged |
| --- | --- | --- | --- | --- | --- | --- |
| EF | SHG | 15 | 9 | 17 | 12 | 13 |
|  | THG | 68 | 65 | 64 | 62 | 65 |

S5. Effective enhancement factor.

Considering that the collected nonlinear signals also include contributions from the background MoS_2_, the actual nonlinear responses of MoS_2_ enhanced by the DoMN should be large. To quantify the real nonlinearity enhanced by the DoMN, we define the effective enhancement factor $\mathrm{EF}_{e}$ as

$\mathrm{EF}_{e}=\frac{I_{\text{dim}}-I_{\text{0}}-I_{\text{dim0}}}{I_{0}}\frac{S_{\text{0}}}{S_{\text{dim}}}$ (5)

where $I_{\text{dim}},$ $I_{\text{0}},$ and $I_{\text{dim0}}$ are the SHG/THG intensities of the MoS_2_-DoMN hybrid structure, pristine monolayer MoS_2_, and the DoMN, respectively. $S_{\text{dim}}$ denotes the area of the dimer (∼0.03μm^2^) and $S_{\text{0}}$ represents the area of MoS_2_ we collected (∼6μm^2^). According to Eq. (5), the enhancement factors are determined to be 3000- and 13600-folds for the SHG and THG of MoS_2_, respectively.

**Table.S2:** Comparisons of THG and SHG $\mathrm{EF}_{e}$.

| **Structure** | **SHG EF_e_** | **THG EF_e_** | **FW/nm** | **Ref.** |
| --- | --- | --- | --- | --- |
| WS_2_/NCOM | 280 | \ | 800 | [4] |
| WSe_2_/trenches | 7000 | \ | 800 | [5] |
| WS_2_/nanogroove | 400 | \ | 860 | [6] |
| WS_2_/nanoantenna | 40 | \ | 1260 | [7] |
| WS_2_/NPoM | 3000 | 3800 | 1240/1260 | [8] |
| MoS_2_/nanohole | 1527 | \ | 869.6 | [9] |
| MoS_2_/NW | 140 | \ | 800 | [10] |
| WSe_2_/NPOM | 3800 | \ | 1450 | [11] |
| MoS_2_(GR)/QD coating | 170 | 60 | 820/1350 | [12] |
| MoS_2_/DoMN | 3000 | 13600 | 1550 | This work |

\ not available.

S6. Polarization-resolved scattering spectra.


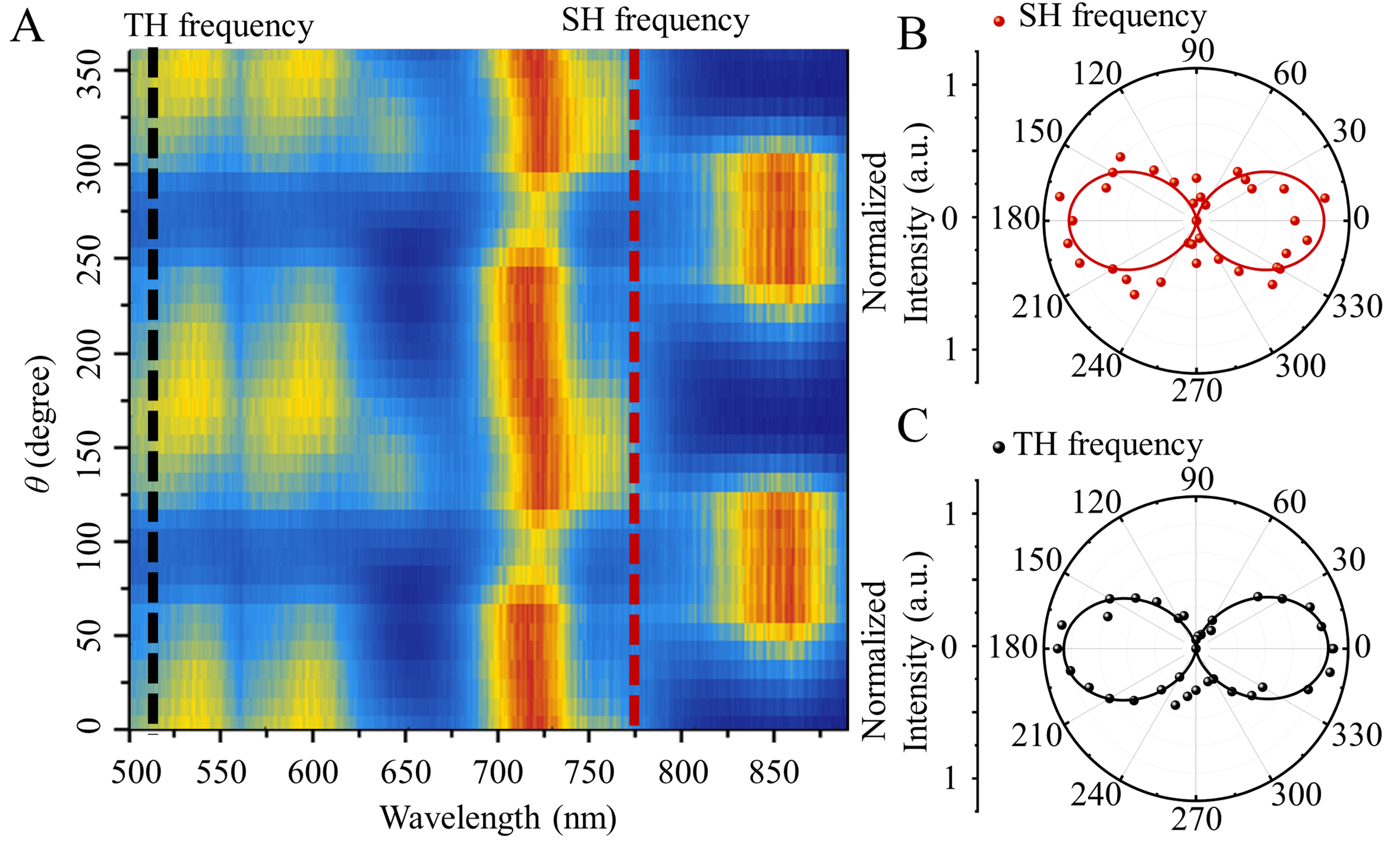


**Figure S4:** (A) Polarization-resolved scattering spectra of MoS_2_-DoMN. The SH and TH frequencies are marked with red and black dashed lines, respectively. Polarization dependence of the (B) SH and (C) TH frequencies, respectively.

S7: SHG far-field amplitude patterns and phase differences (|*ϕ*_MoS2_-*ϕ*_DoMN_|) of the SHG far-field between MoS_2_ and DoMN in the *xz*-plane.

**
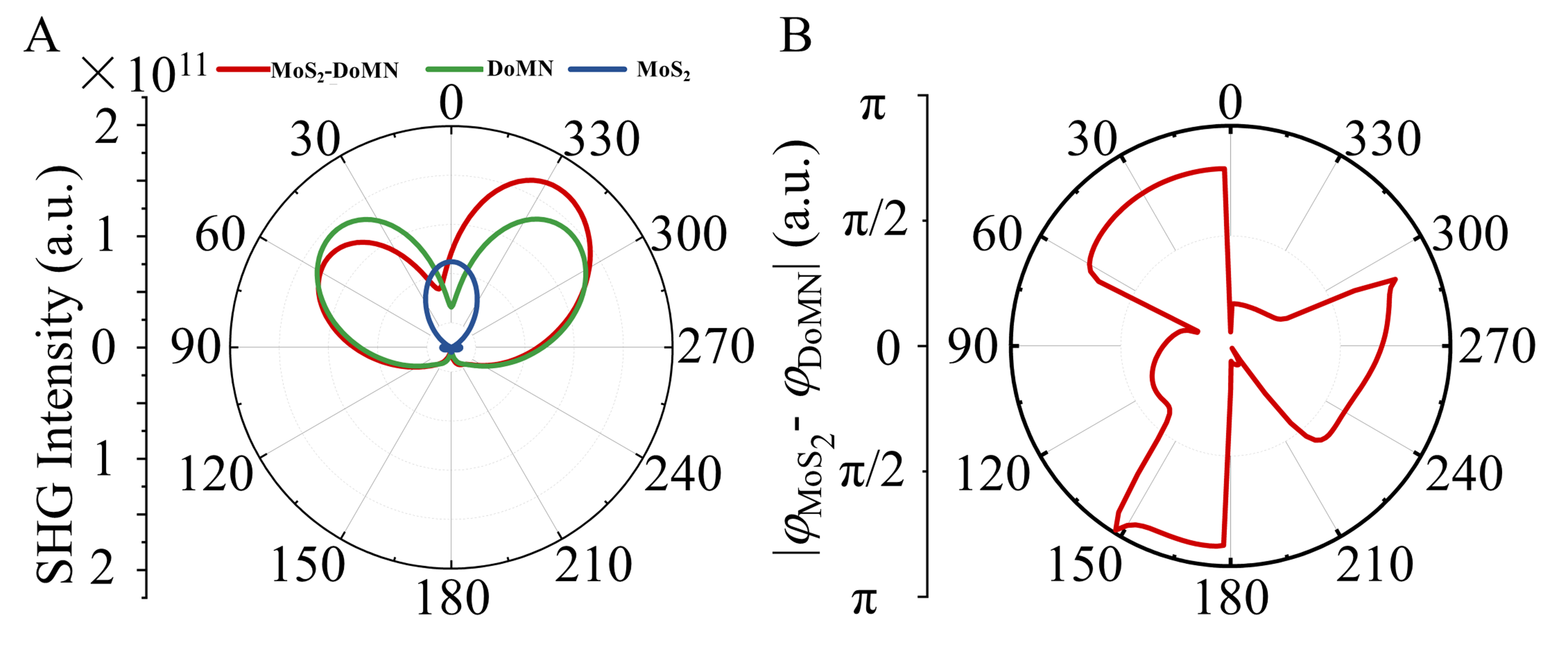
**

**Figure S5:** (A) SHG far-field amplitude patterns of the monolayer MoS_2_ (blue solid curves), the DoMN (green solid curves) and the MoS_2_-DoMN (red solid curves) in the *xz*-plane. (B) Phase differences (|*ϕ*_MoS2_-*ϕ*_DoMN_|) of the SHG far-field between MoS_2_ and DoMN in the *xz*-plane.

S8: Calculation of the parallel components of the SHG and THG intensities

The polarization dependence of SHG for pristine MoS_2_ and MoS_2_-DoMN in Figure 3(E) can be described as [6]:

$I_{\mathrm{MoS}_{2}}^{(2)}\propto\left[ \chi_{yyy}^{\left( 2 \right)}cos(2\theta+3\theta_{0}) \right]^{2}$ (6)

and

$I^{(2)}\propto\left[ \chi_{yyy}^{\left( 2 \right)}\left( \text{cos}^{2}\theta-c_{0}\text{sin}^{2}\theta\right)\text{cos3}\theta_{0}-c_{0}\text{sin2}\theta\text{sin}3\theta_{0} \right]$ (7)

where $c_{0}$ is the coupling coefficient of the pump laser with the dimer, $\theta$ is the angle between the pump laser and the dimer long axis and $\theta_{0}$ is the mismatch angle between the armchair ($\gamma$) direction and the dimer axis (about 3.13° for the measured sample).

The parallel components THG intensities of the pristine MoS_2_ and MoS_2_-DoMN in Figure 3(F) could be described as:

$I_{\mathrm{MoS}_{2}}^{(3)}\propto\left[ A\sin\left( \theta+2\theta_{0} \right)+Bsin(\theta+\theta_{0}) \right]^{2}$ (8)

and

$\text{I}^{(3)}\propto\left[ A\left( \text{cos}^{2}\theta+{c_{0}}^{2}\text{sin}^{2}\theta\right)\cos\theta+B\cos\theta+C\sin\left( \theta+\theta_{0} \right) \right]^{2}$ (9)

where $A$*,* $B$ and $C$ contain all of the nonzero elements in the third-order susceptibility tensor $(\chi_{yyyy}^{\left( 3 \right)})$.

**References**

[1] A. Saynatjoki, L. Karvonen, H. Rostami, A. Autere, S. Mehravar, A. Lombardo, R. A. Norwood, T. Hasan, N. Peyghambarian, H. Lipsanen, K. Kieu, A. C. Ferrari, M. Polini, and Z. Sun, "Ultra-strong nonlinear optical processes and trigonal warping in MoS_2_ layers," *Nat. Commun.,* vol. 8, no. 1, pp. 893, 2017.

[2] Y. Uchiho, M. Shimojo, and K. Kajikawa, "Electro-optic effect and optical rectification in gold nanoparticles immobilized above a gold surface," *J. Phys. D: Appl. Phys.,* vol. 43, pp. 495101, 2010.

[3] D. Compton, L. Cornish, and E. v. d. Lingen, "The third order nonlinear optical properties of gold nanoparticles in glasses, part II," *Gold Bull.,* vol. 36, no. 2, pp. 51-58, 2003.

[4] X. Han, K. Wang, P. D. Persaud, X. Xing, W. Liu, H. Long, F. Li, B. Wang, M. R. Singh, and P. Lu, "Harmonic resonance enhanced second-harmonic generation in the monolayer WS_2_-Ag nanocavity," *ACS Photonics,* vol. 7, no. 3, pp. 562-568, 2020.

[5] Z. Wang, Z. Dong, H. Zhu, L. Jin, M. H. Chiu, L. J. Li, Q. H. Xu, G. Eda, S. A. Maier, A. T. S. Wee, C. W. Qiu, and J. K. W. Yang, "Selectively plasmon-enhanced second-harmonic generation from monolayer tungsten diselenide on flexible substrates," *ACS Nano,* vol. 12, no. 2, pp. 1859-1867, 2018.

[6] J. Shi, W.-Y. Liang, S. S. Raja, Y. Sang, X.-Q. Zhang, C.-A. Chen, Y. Wang, X. Yang, Y.-H. Lee, H. Ahn, and S. Gwo, "Plasmonic enhancement and manipulation of optical nonlinearity in monolayer tungsten disulfide," *Laser Photonics Rev.,* vol. 12, no. 10, pp. 1800188, 2018.

[7] F. Spreyer, C. Ruppert, P. Georgi, and T. Zentgraf, "Influence of plasmon resonances and symmetry effects on second harmonic generation in WS_2_-plasmonic hybrid metasurfaces," *ACS Nano,* vol. 15, no. 10, pp. 16719-16728, 2021.

[8] J. Shi, Z. Lin, Z. Zhu, J. Zhou, G. Q. Xu, and Q. H. Xu, "Probing excitonic rydberg states by plasmon enhanced nonlinear optical spectroscopy in monolayer WS_2_ at room temperature," *ACS Nano,* vol. 16, no. 10, pp. 15862-15872, 2022.

[9] Q. Leng, H. Su, J. Liu, L. Zhou, K. Qin, Q. Wang, J. Fu, S. Wu, and X. Zhang, "Enhanced second-harmonic generation in monolayer MoS_2_ on suspended metallic nanostructures by plasmonic resonances," *Nanophotonics,* vol. 10, no. 7, pp. 1871-1877, 2021.

[10] D. Li, C. Wei, J. Song, X. Huang, F. Wang, K. Liu, W. Xiong, X. Hong, B. Cui, A. Feng, L. Jiang, and Y. Lu, "Anisotropic enhancement of second-harmonic generation in monolayer and bilayer MoS_2_ by integrating with TiO_2_ nanowires," *Nano Lett.,* vol. 19, no. 6, pp. 4195-4204, 2019.

[11] T. Zhang, Q. Guo, Z. Shi, S. Zhang, and H. Xu, "Coherent second harmonic generation enhanced by coherent plasmon-exciton coupling in plasmonic nanocavities," *ACS Photonics,* vol. 10, no. 5, pp. 1529-1537, 2023.

[12] H. Hong, C. Wu, Z. Zhao, Y. Zuo, J. Wang, C. Liu, J. Zhang, F. Wang, J. Feng, H. Shen, J. Yin, Y. Wu, Y. Zhao, K. Liu, P. Gao, S. Meng, S. Wu, Z. Sun, K. Liu, and J. Xiong, "Giant enhancement of optical nonlinearity in two-dimensional materials by multiphoton-excitation resonance energy transfer from quantum dots," *Nat. Photonics,* vol. 15, no. 7, pp. 510-515, 2021.
